# Comparison of Cryoballoon Ablation Methods in Pulmonary Vein Isolation

**DOI:** 10.3390/medicina61111920

**Published:** 2025-10-26

**Authors:** Kaspars Kupics, Raivis Bricis, Kristine Jubele, Ieva Ansaberga, Oskars Kalējs, Andrejs Erglis

**Affiliations:** 1 Department of Internal Diseases, Pauls Stradins Clinical University Hospital, 13 Pilsoņu Str., LV-1002 Rīga, Latvia; kristine.jubele@stradini.lv (K.J.); ieva.ansaberga@stradini.lv (I.A.); oskars.kalejs@stradini.lv (O.K.); a.a.erglis@stradini.lv (A.E.); 2 Faculty of Medicine, Rīga Stradins University, 16 Dzirciema Str., LV-1007 Rīga, Latvia; 3 Department of Internal Diseases, Faculty of Medicine, Rīga Stradins University, 16 Dzirciema Str., LV-1007 Rīga, Latvia; 4 Institute of Cardiology and Regenerative Medicine, 13 Pilsoņu Str., LV-1002 Rīga, Latvia

**Keywords:** cryoballoon ablation, atrial fibrillation, pulmonary vein isolation

## Abstract

*Background and Objectives:* Cryoballoon ablation is a well-established therapy for atrial fibrillation (AF), enabling pulmonary vein isolation (PVI) using a single-shot technique. The two primary systems—Medtronic Arctic Front and the newer Boston Scientific POLARx—differ in design and performance characteristics, but few direct comparisons exist. This study aimed to compare the biophysical parameters and mid-term outcomes of the POLARx and Arctic Front cryoballoon systems. *Materials and Methods:* In a retrospective analysis of 200 patients who underwent cryoballoon ablation for paroxysmal or persistent AF, patients were grouped by ablation system: POLARx (*n* = 107) and Arctic Front (*n* = 93). Key parameters including nadir balloon temperatures, time to reach −40 °C, procedure duration, dose area product (DAP), complication rates, and recurrence of AF were assessed. *Results:* The POLARx system achieved significantly lower nadir temperatures in all pulmonary veins compared to Arctic Front (left superior PV: −57.6 ± 5.0 °C vs. −50.1 ± 5.7 °C, *p* < 0.001). Time to reach −40 °C was also shorter with POLARx (left superior PV: 23.3 ± 7.3 s vs. 33.3 ± 11.5 s, *p* < 0.001). Despite these advantages, procedure time was longer in the POLARx group (64.7 ± 14.8 min vs. 51.6 ± 19.7 min, *p* < 0.001). AF recurrence at 11.8 months was similar (33.6% in POLARx vs. 39.8% in Arctic Front, *p* = 0.93). Phrenic nerve palsy occurred in 5.0% (POLARx) and 4.3% (Arctic Front), with no cases of cardiac tamponade. *Conclusions:* While both systems demonstrated similar efficacy and safety, POLARx was associated with superior cooling kinetics and biophysical performance.

## 1. Introduction

Atrial fibrillation (AF) is a supraventricular arrythmia with uncoordinated atrial activation that results in a loss of effective atrial contraction [1]. It is the most common sustained cardiac arrythmia worldwide and is associated with a substantial increase in morbidity and mortality due to complications such as systemic thromboembolism, stroke, and heart failure [2,3]. The most common types of AF include paroxysmal AF, which is defined as AF which terminates spontaneously within 7 days or with the assistance of an intervention, and persistent AF, which are not self-terminating within a 7-day period [1].

Approximately 2% of the world’s population is affected by AF, with the highest incidence and prevalence in people aged 75 and above, regardless of gender [2]. From 2010 until 2019, the global prevalence of AF grew from 33.5 million to 59 million people [2]. In 2021, AF affected 52.55 million people worldwide, representing a 137% increase since 1990 [4].

First-line therapy for paroxysmal AF includes strategies for symptom relief and prevention of serious adverse events through rhythm control, heart rate control, and anticoagulation medication [1,5]. Catheter ablation is a Class I recommendation for patients with symptomatic paroxysmal AF in whom antiarrhythmic drugs (AADs) are ineffective or not tolerated, as well as the first-line treatment option opposed to AADs [1,6]. For patients with persistent AF, catheter ablation is a Class IIb recommendation, and it is recommended only when antiarrhythmic drug therapy has failed [5]. Recent randomized trials have also shown that ablation can be a viable initial treatment choice to drug therapy, as it has a significantly lower AF recurrence rate, lower symptom recurrence, and a similar rate of serious adverse events [6,7,8,9].

At the moment, the non-drug-related treatment available includes radiofrequency, cryoballoon, and pulse wave ablation. As pulse field technology advances, many procedures continue to rely on the cryoballoon method due to its relative simplicity and reproducibility [10,11,12]. Compared to radiofrequency ablation, cryoballoon therapy enables the single-shot isolation of the pulmonary vein in a reproducible and technically straightforward manner [10,13]. As such, it is less dependent on various factors such as operator experience and technical skills; this has been demonstrated in various meta-analyses and confirms its non-inferiority in defined patient cohorts [3,11].

The leading providers of cryoballoon ablation are Boston Scientific, with POLARx, and Medtronic, with Arctic Front machines. Arctic Front has been used since 2010 and has been the primary machine for cryoballoon ablation for decades, being used in landmark studies such as FIRE AND ICE and STOP AF [10,12]. POLARx is a relatively new method, having been approved in Europe in 2020 and in the USA in August 2023. The latter of the two machines allows for a more stable pressure and size of the balloon than the former, creating a more uniform pulmonary vein lesion [14,15,16]. The differences between these systems are therefore susceptible to different procedural characteristics such as dosing time and balloon temperature [17,18,19].

Increased attention is paid to the diagnostic value of biophysical feedback during ablation, such as nadir temperature and time to reach certain temperature thresholds. These can be used to assess successful isolations and to predict lesion durability and long-term freedom from AF [20,21]. Further understanding of these parameters could allow for optimization of the procedure, such as minimizing the occurrence of complications such as phrenic nerve palsy or esophageal injury [20,22,23].

This research aims to compare the biophysical parameters of Boston Scientific and Medtronic machines, as well as procedural metrics and clinical outcomes that may further portray the impact of the differences between both technologies on procedural diagnostics and treatment outcomes. Only a few published papers have compared the two machines’ clinical outcomes [24,25]. More research is needed to accomplish this, as most of the data is not consistent [17,26,27]. The most extensive research into this topic is being conducted by the Frozen-AF trial in the United States of America [16].

## 2. Materials and Methods

This retrospective, single-center study analyzed patients who underwent cryoballoon ablation for paroxysmal or persistent atrial fibrillation (AF) at Pauls Stradins Clinical University Hospital. Two hundred patients who underwent cryoballoon ablation between January of 2020 and December of 2023 were included in this study. All consecutive eligible patients treated during the study period were included; no formal sample size calculation was performed due to the exploratory design of the study. The patients were randomly divided into two cohorts based on the Medtronic Arctic Front^TM^ (Medtronic, Minneapolis, MN, USA) or Boston Scientific POLARx^TM^ machine (Boston Scientific, Marlborough, MA, USA). The 200 patients included were between the ages of 31 and 80 years and had symptomatic AF, including both paroxysmal and persistent AF. The patients had a follow-up visit where they were assessed for recurrent AF or other rhythm dysfunction, and the duration of arrythmia-free aurvival after the procedure was evaluated. The follow-up was performed 9–12 months after cryoballoon ablation. Recurrence was defined as symptomatic AF episodes reported after a 90-day blanking period.

This study was approved by the local ethics committee on 22 April 2025. The design and reporting of this study followed the STROBE guidelines as recommended by the EQUATOR network. Patients with incomplete procedural data and contraindications were not included in the study.

### 2.1. Statistical Data Analysis

Continuous variables were assessed for normality using the Shapiro–Wilk test. Normally distributed variables are presented as means *±* standard deviations and were compared using an independent-samples *t*-test. Skewed variables such as DAP are presented as medians (interquartile ranges, IQRs) and were compared using the Mann–Whitney U test. Categorical variables were presented as n (%), compared using χ^2^ or Fisher’s exact test, as appropriate. *p* < 0.05 was considered statistically significant.

Data analysis was performed using IBM SPSS Statistics 30.0 (IBM Corp., Armonk, NY, USA). The graphs were created using Graphpad Prism 10 (GraphPad Software, San Diego, CA, USA). Data on the patients was gathered from Pauls Stradins Clinical University Hospital’s cryoballoon ablation procedure protocol, which gives information on procedural characteristics such as the temperature of the balloon and the time required to reach said temperature. The cryoballoon ablation protocol used in this study was consistent with current international practice and aligns with recommendations later formalized in the 2024 European Heart Rhythm Association/Heart Rhythm Society/Asia Pacific Heart Rhythm Society/Latin American Heart Rhythm Society expert consensus statement. Data collection was retrospective.

### 2.2. Procedure Description

The patients were admitted the day before the procedure. All were on direct oral anticoagulants or warfarin therapy, with international normalized ratio (INR) between 2 and 3. The last dose of the direct oral anticoagulants was administered the evening before the procedure if used twice per day and on the morning of the previous day if administered once per day. Patients on warfarin were monitored to ensure that their INR level on the day of the procedure was as close to 2 as possible. Antiarrhythmic medications were not stopped before the operation. Procedures were performed in an electrophysiological cath lab under general anesthesia. For each patient, specific periprocedural data were collected using procedural data sheaths. Electrophysiological recordings were performed using the CardioLab system (Prucka, GE Healthcare, Chicago, IL, USA). All the pacing maneuvers were performed using the MicroPace system (MicroPace EP Inc., Santa Ana, CA, USA). Thrombi in the atria were evaluated by transesophageal echocardiography. All procedures were performed by the same experienced electrophysiology team familiar with both systems, thus minimizing operator variability.

After gaining right-sided femoral vein access, two introducers (6 French (Fr) and 8 Fr) were placed. Then, a 6 Fr 10-pole coronary sinus catheter was introduced into the coronary sinus. Afterwards, a 110 cm wire 0.037 Fr was located in the superior cava vein. Then, the long, steerable cryoablation sheath advanced over the wire, and after the wire was removed, a 96 cm transeptal needle was introduced. Atrial septal puncture was performed using the cryoablation sheath and needle directly under X-ray, contrast dye, and transesophageal echocardiography control. Immediately after the puncture, weight-adjusted heparin was administered, and activated clotting time (ACT) was monitored throughout the procedure, maintaining an ACT level above 350 s.

Following the puncture, the previously prepared cryoablation balloon was inserted into the left atrium, and the circular mapping catheter was located in one of the pulmonary veins. All the pulmonary vein signals were recorded and annotated. For the Medtronic and Boston cryoballoon catheter, a 28 mm size was used. Cryoablation was performed after sealing the vein ostium with the balloon and confirming the seal with contrast dye. During vein sealing, a circular catheter was placed as close as possible to the ostia of the veins, and pulmonary vein atrial muscle sleeve signals were recorded. Per protocol, at the operator’s discretion, freezing times per vein varied from 180 s to 240 s or longer. The ablation protocol was designed for a single application per vein, provided the isolation time was less than 40 s and the minimal freezing temperature was below 40 degrees Celsius. Additional applications were conducted if the above criteria were not met. The circular catheter recordings of the pulmonary veins confirmed pulmonary vein isolation. Performing right-sided vein isolation, a coronary sinus catheter was placed in the superior vena cava and paced to ensure the phrenic nerve capture. Pacing-induced right-sided diaphragm muscle contraction was evaluated by manual diaphragm movement palpation during ablation. If the sensation of the diaphragm excursions was diminishing, ablation was immediately stopped, and the balloon deflated.

After the last pulmonary vein was isolated, all the previous ones were checked, and isolation was confirmed. The entrance and exit block confirmed pulmonary vein isolation. After the vein isolation was confirmed, the sheath was withdrawn, and the puncture site was closed by a figure-of-eight suture. No protamine sulfate was given at the end of the operation. Anticoagulants were re-administered 4 to 6 h after the procedure. Patients were discharged the next day, and follow-up visits were planned. Post-discharge treatment was ensured to be as close as possible to the existing European Society of Cardiology guidelines.

## 3. Results

### Procedural Data Analysis

Patients were divided into two groups based on the machine used, resulting in an approximately 1:1 ratio, which created two distinct groups. The POLARx group comprised 107 patients, while the Arctic Front group comprised 93 patients. Further detail is shown in Table 1.

At the beginning of the procedure, a total of 108 patients had sinus rhythm (54%), and 53 patients had AF (26.5%), while the rhythm prior to the procedure was not reported in 39 patients (19.5%), *p* = 0.901. Thirty-six patients from the Arctic Front (18%) group and sixty-eight patients from the POLARx (34%) group needed periprocedural cardioversion, *p* = 0.396. After the ablation procedure, 158 patients (79%) had sinus rhythm, while only 2 (1%) had AF directly after the procedure, *p* < 0.001. The procedure time for POLARx, on average, was 64.7 ± 14.8 min, and for Arctic Front, it was 51.6 ± 19.7 min, *p* < 0.001. The radiation time for the POLARx group was 10.9 ± 5.7 min, and for the Arctic Front group, it was 10.6 ± 4.6 min, *p* = 0.621. The cumulative dose area product (DAP) for Boston Scientific was 788.9 ± 114.4, and for Artic Front, it was 1056.2 ± 1386.4, *p* = 0.143. Radiation time between both types of the procedure was not statistically significant (t(195.6) = 0.5, *p* = 0.615), while procedure time was statistically significant (t(173) = 5.01, *p* < 0.001).

Twelve patients had a common ostium, with eleven in the PolarX group and one in the Arctic Front group, *p* = 0.983. The right inferior vein is the most challenging of the pulmonary veins, requiring the operator to perform various techniques, such as a hockey-stick and pull-down maneuver [15]. In the Arctic Front group, this observation was consistent as the right inferior pulmonary vein (RIPV) had the most additional ablations (28), followed by the left superior pulmonary vein (LSPV) (24), left inferior pulmonary vein (LIPV) (18), and right superior pulmonary vein (RSPV) (15). However, in the POLARx group, the vein with most additional ablations was LSPV (27), followed by LIPV (23), RIPV (18), and RSPV (16).

Overall, there were 117 patients who needed additional applications during the procedure, 60 of whom were in the POLARx group (30%) and 57 in the Arctic Front group (28.5%), *p* = 0.458. In the case of Arctic Front, only eight patients had n. phrenicus paresis (4%), while the POLARx group had ten such cases (5%), *p* = 0,34. After the follow-up (average time: 11.8 months), 36 patients from the POLARx group complained of having recurrent AF (18%), while 37 patients from the Arctic Front had the same complaints (18.5%), *p* = 0.177. ACT with POLARx was 359.7 ± 46.8, while with Arctic Front, it was 355.5 ± 54.2, *p* = 0.554. No tamponades were observed during the periprocedural time.

**Figure 1:**
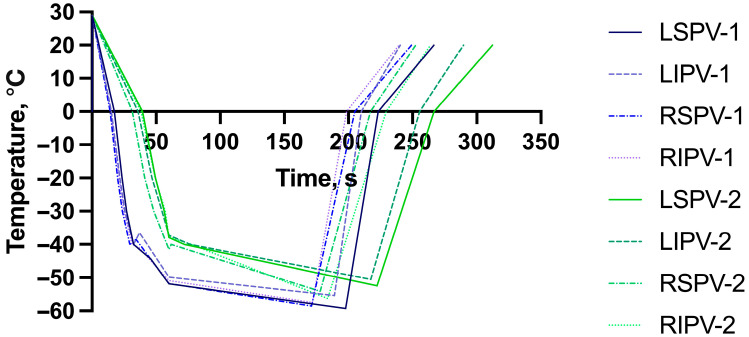
Freezing characteristics of each pulmonary vein on a time–temperature graph. The blue-colored lines refer to Boston Scientific POLARx (labeled as 1), while the green-colored lines refer to Medtronic Arctic Front (labeled as 2). LSPV—left superior pulmonary vein; LIPV—left inferior pulmonary vein; RSPV—right superior pulmonary vein; RIPV—right inferior pulmonary vein. The POLARx system achieved a steeper temperature drop within a shorter time than the Arctic Front system frame across all veins within the same freezing duration, reflecting quicker cooling dynamics.

The biophysical parameters measured for each vein are shown in Table 2 and in Figure 1.

Table 2 demonstrates consistently faster cooling dynamics with the POLARx system compared with Arctic Front. In all pulmonary veins, POLARx achieved 0 °C and −40 °C significantly sooner and reached lower nadir temperatures within shorter freeze times. Rewarming times to 0 °C were also shorter, indicating more efficient thermal exchange. Despite these temperature and timing differences, isolation times and isolation temperatures were largely comparable between the systems, suggesting that both achieve effective vein isolation. Clinically, the faster and deeper cooling of the POLARx balloon may translate into more uniform lesion formation and shorter overall procedure times without compromising efficacy or safety.

## 4. Discussion

Consistent with previous reports, such as Knappe et al. (2025), the POLARx system demonstrated lower intraprocedural nadir temperatures than Arctic Front in all four pulmonary veins [18,28]. This is likely due to the temperature probe’s location and balloon design differences. Despite these lower temperatures, the incidence of phrenic nerve palsy remained similar between the two systems (5% in POLARx vs. 4% in Arctic Front, *p* = 0.34), supporting the notion that temperature alone does not determine safety outcomes; rather, they are influenced by a combination of anatomical factors, technique, and device control mechanisms [20,26].

Despite achieving lower temperatures and faster time-to-cooling benchmarks, the POLARx group required longer procedural durations (64.7 ± 14.8 min vs. 51.6 ± 19.7 min, *p* < 0.001). This may be attributed to the increased need for catheter repositioning or challenges associated with the newer system’s learning curve [16]. While POLARx offers favorable energy delivery, early operator experience may influence outcomes during the technology transition phase [29]. The longer procedure time with POLARx was also reported in other studies, including the Frozen-AF trial, suggesting that operator familiarity plays a significant role in procedural efficiency [16].

As measured by cumulative DAP, radiation exposure was lower in the POLARx group (788.9 ± 114.4 vs. 1056.2 ± 1386.4 mGy*cm^2^), although the difference was not statistically significant. These findings align with prior data showing that both systems can be optimized for reduced radiation when guided by procedural strategies like SWEET-Cryo [23]. The similar fluoroscopy times reinforce the conclusion that the significant difference in DAP may be related more to imaging settings or patient-specific anatomy than to system capabilities.

Clinical effectiveness, defined by freedom from atrial fibrillation at follow-up (average 11.8 months), was also similar between the two groups (33.6% recurrence in POLARx vs. 39.8% in Arctic Front). This aligns with data from the FROZEN-AF and COMPARE CRYO trials, which also showed no significant differences in arrhythmia recurrence between cryoballoon systems when procedural endpoints were achieved [16,27]. From a biophysical point of view, POLARx demonstrated faster cooling dynamics, significantly shorter times at 0 ° C and −40 ° C, and colder temperatures at 60 s across all veins. This rapid cooling could theoretically improve lesion durability, although it has not yet translated into superior clinical outcomes in mid-term follow-up [21,26,27].

Of particular note, the RIPV was consistently the most difficult to isolate, requiring advanced manipulation regardless of the system used. However, operators subjectively reported that the greater flexibility of the POLARx sheath aided in navigating challenging anatomy, which was also reflected in the literature by Moser et al. (2022), Su et al. (2018), and the ICE-AGE-X study [20,22,30]. Our results confirmed that the extra flexibility of the Boston POLARx sheath is helpful during right inferior pulmonary vein isolation, as unlike the Medtronic Arctic Front sheath, ablation for this vein was performed less often.

Recent studies have underlined the importance of using biophysical markers not just for procedural feedback but also for intraoperative diagnostics. Aryana et al. (2016) emphasized that variables like time-to-isolation, temperature slopes, and rewarming time can be associated with lesion durability and long-term success [20,21,31]. Incorporating these markers into procedural strategy may allow operators to identify inadequate applications in real time.

As studies like Su et al. (2018), Tomaiko-Clark et al. (2022), and Hachem et al. (2018) point out, consistent performance metrics across various anatomical contexts are essential to ensure reproducibility across centers and operators [3,17,20]. In this study, although the POLARx demonstrated superior technical precision in terms of temperature control, its benefit in difficult-to-isolate veins did not lead to significantly improved clinical outcomes. This reinforces the idea that while biophysical markers are valuable, they are only part of a broader procedural success framework.

In terms of safety, the absence of tamponades and low complication rates in both groups support the favorable safety profile of cryoballoon ablation overall. Nevertheless, further evaluation of rare but serious complications would require larger, multicenter cohorts.

### Strengths and Limitations

In terms of strengths, the study offers a well-defined cohort, standardized procedure, and a direct comparison of two widely used cryoballoon ablation systems.

Regarding limitations, there are several limitations to this study. First, the retrospective design may have introduced selection and recall bias, as patients were not randomized to cryoballoon systems. Although baseline characteristics between groups were generally balanced, unknown confounders and reliance on previously recorded data cannot be ruled out. However, all consecutive cases within the study period were included to minimize potential selection bias. Second, the follow-up period (9–12 months) may not fully capture late AF recurrence, which can occur even after apparently successful initial ablation [10,32]. Third, the study was conducted at a single center, limiting generalizability to broader populations and practice settings. Fourth, the cryoballoon ablation protocol is limited in terms of information available. Therefore, the comorbidities of the patients and precise medications are not mentioned in Table 1 with the rest of baseline characteristics.

## 5. Conclusions

In this retrospective study, POLARx cryoablation resulted in patient biophysical parameters and a number of repeated post-operative AF episodes that were non-inferior to those achieved by Arctic Front cryoablation.

Future multicenter, prospective, randomized trials with standardized monitoring protocols and longer follow-up are necessary to validate these findings.

## Figures and Tables

**Table 1:** Baseline characteristics of study population.

	POLARx	Arctic Front	*p*-Value
Age	62.3 ± 10.1	61.4 ± 10.2	0.254
Male gender	42 (39.3)	35 (37.6)	
BMI, kg/m^2^	29.94 ± 5.29	31.58 ± 5.82	0.041
LAVI, mL/m^2^	35.9 ± 8.1	35.5 ± 8.8	0.676
CHA_2_DS_2_VASC	1.9 ± 1.7	1.7 ± 1.6	0.881
0	19	27	
1	18	22	
2	26	19	
3	16	13	
4	1	8	
5	4	2	
6	1	0	
7	2	2	
Use of AADs	85 (79.4)	71 (76.3)	
EHRA	2.4 ± 0.7	2.5 ± 0.7	0.546

Values are mean ± standard deviation. BMI—body mass index; LAVI—left atrial volume index; CHA_2_DS_2_VASC—CHA2DS_2_-VASc score; AAD—antiarrhythmic drugs; EHRA—European Heart Rhythm Association score of atrial fibrillation.

**Table 2:** Procedural characteristics from cryoballoon ablation protocol.

	POLARx	Arctic Front	*p* Value
LSPV FT, s	197.8 ± 37.7	222.3 ± 32.6	<0.001
LSPV 0 °C, s	17.3 ± 1.7	39.5 ± 15.1	<0.001
LSPV −40 °C, s	32.5 ± 3.8	72.1 ± 24.5	<0.001
LSPV temp at 60 s	−51.9 ± 4.6	−37.9 ± 15.5	<0.001
LSPV isolation time, s	45.8 ± 22.1	48.7 ± 39.4	0.578
LSPV isolation temperature, °C	−44.4 ± 9.8	−37.3 ± 16.3	0.002
LSPV minimal temperature, °C	−59.3 ± 5.8	−52.5 ± 19.1	<0.001
LSPV RT to 0 °C, s	223.3 ± 38.5	269.9 ± 39.2	<0.001
LIPV FT, s	189.2 ± 34.4	217.5 ± 38.0	<0.001
LIPV 0 °C, s	15.1 ± 1.9	35.8 ± 14.0	<0.001
LIPV −40 °C, s	32.3 ± 4.4	76.5 ± 29.5	<0.001
LIPV temp at 60 s	−49.9 ± 4.4	−37.3 ± 12.3	<0.001
LIPV isolation time, s	37.0 ± 25.0	41.8 ± 26.7	0.286
LIPV isolation temperature, °C	−36.5 ± 13.3	−33.7 ± 11.7	0.328
LIPV minimal temperature, °C	−55.4 ± 5.7	−50.5 ± 20.8	0.034
LIPV RT to 0 °C, s	210.0 ± 36.5	255.6 ± 42.9	<0.001
RSPV FT, s	171.2 ± 34.3	178.0 ± 20.5	0.102
RSPV 0 °C, s	14.0 ± 1.5	31.5 ± 11.2	<0.001
RSPV −40 °C, s	29.5 ± 5.4	61.7 ± 19.5	<0.001
RSPV temp at 60 s	−51.8 ± 11.4	−41.6 ± 12.6	<0.001
RSPV isolation time, s	34.3 ± 18.9	31.0 ± 17.7	0.290
RSPV isolation temperature, °C	−38.5 ± 11.0	−31.1 ± 12.0	<0.001
RSPV minimal temperature, °C	−58.6 ± 13.4	−54.2 ± 14.4	0.028
RSPV RT to 0 °C, s	205.1 ± 23.7	217.3 ± 28.7	0.002
RIPV FT, s	172.1 ± 30.8	183.8 ± 33.4	0.011
RIPV 0 °C, s	14.6 ± 1.4	38.1 ± 19.0	<0.001
RIPV −40 °C, s	31.3 ± 4.2	77.7 ± 29.4	<0.001
RIPV temp at 60 s	−50.9 ± 5.0	−37.8 ± 12.1	<0.001
RIPV isolation time, s	36.6 ± 14.2	47.2 ± 30.0	0.008
RIPV isolation temperature, °C	−40.9 ± 9.9	−37.7 ± 10.8	0.075
RIPV minimal temperature, °C	−57.5 ± 6.6	−56.3 ± 42.7	0.768
RIPV RT to 0 °C, s	198.7 ± 22.7	229.6 ± 33.8	<0.001

Values are mean ± standard deviation. LSPV—left superior pulmonary vein; LIPV—left inferior pulmonary vein; RSPV—right superior pulmonary vein; RIPV—right inferior pulmonary vein; FT—freeze time; RT—rewarming time; s—seconds.

## Data Availability

The data that support the findings of this study are available from the corresponding author upon reasonable request.
